# A Rare Case of Gastrointestinal Amyloidosis Presenting as Dysphagia

**DOI:** 10.7759/cureus.22085

**Published:** 2022-02-10

**Authors:** Shaina Ailawadi, Bahar K Cheema, Syed Salahuddin, Sangeeta Agrawal

**Affiliations:** 1 Internal Medicine, Wright State University Boonshoft School of Medicine, Dayton, USA; 2 Department of Gastroenterology, Wright State University Boonshoft School of Medicine, Dayton, USA; 3 Department of Pathology, Dayton Veterans Affairs Medical Center, Dayton, USA

**Keywords:** al amyloid, endoscopy, gastric amyloidosis, esophageal amyloidosis, congo red, esophagogastroduodenoscopy (egd), dysphagia

## Abstract

A 74-year-old male with an unintentional 20-pound weight loss over the past year presented with new-onset dysphagia to solid foods. Esophagogastroduodenoscopy showed a subtle stricture with ill-defined scar tissue-like findings in the distal esophagus and erosions in the antrum of the stomach without masses. Pathologic findings showed the presence of amyloidosis involving the proximal and distal esophagus, as well as gastric mucosa with chronic inflammation and reactive epithelial changes. We present a rare case of dysphagia as the initial presentation of gastrointestinal amyloidosis.

## Introduction

Amyloidosis is characterized by the extracellular tissue deposition of insoluble protein subunits known as fibrils [1]. These fibrils are resistant to normal proteolytic degradation and often deposit and replace normal tissue within several organ systems, leading to a large variety of pathological presentations [2]. Furthermore, the numerous fibril types, locations, and extent of deposition generate variable clinical manifestations leading to significant diagnostic and treatment challenges [1,3]. Specifically, the deposition of these abnormal proteins within the gastrointestinal (GI) tract interferes with the GI organ structure and function, most commonly in the liver and small bowel, presenting as cirrhotic sequelae, malabsorption, and GI bleeding [4-6]. Amyloidosis with esophageal involvement has been reported to vary among GI involvement from 13% in a radiological study to 22% in an autopsy series, with the most common symptomatic presentation being gastroesophageal reflux. However, the presentation of dysphagia secondary to amyloidosis involvement is an uncommon finding [5]. This case report demonstrates a 74-year-old male who presented with a one-month history of dysphagia to solid food with a peculiar finding on esophagogastroduodenoscopy (EGD), leading to the rare diagnosis of esophageal and gastric amyloidosis. 

## Case presentation

**Figure 1:**
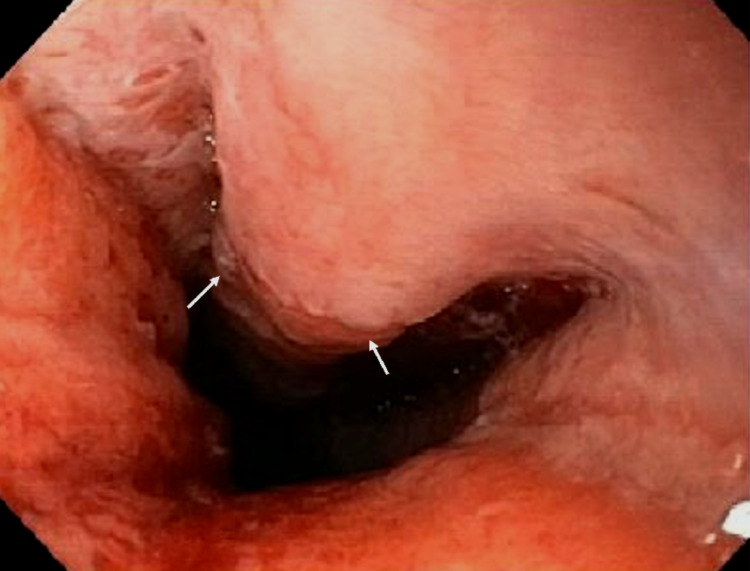
Endoscopic image of the distal esophagus showing a stricture.

**Figure 2:**
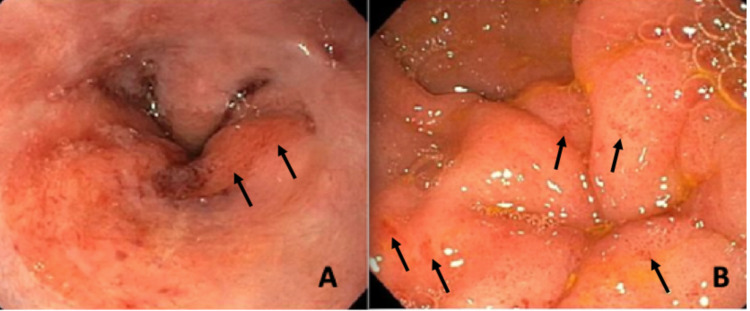
Endoscopic images of the (A) gastroesophageal junction showing a scar tissue-like area and (B) erosions in the antrum of the stomach with no evidence of masses.

Our patient was a 74-year-old male with a medical history of congestive heart failure, hyperlipidemia, hypertension, and anemia, presenting with heart failure exacerbation and an unintentional 20-pound weight loss over the past year. The patient reported new-onset dysphagia to solid foods in the last month without odynophagia. As per inpatient speech pathology evaluation, the patient had significant oropharyngeal dysphagia with overt signs of aspiration of solid foods. Laboratory test results were significant for elevated creatinine of 2 mg/dL (baseline 1.4 mg/dL), elevated urine protein, elevated free kappa light chains, and elevated free lambda light chains with normal kappa/lambda ratio. The EGD findings in the distal esophagus showed an obscure stricture (Figure 1) with a scar tissue-like area noted near the gastroesophageal junction (Figure 2A) and erosions in the antrum of the stomach with no evidence of masses (Figure 2B).

**Figure 3:**
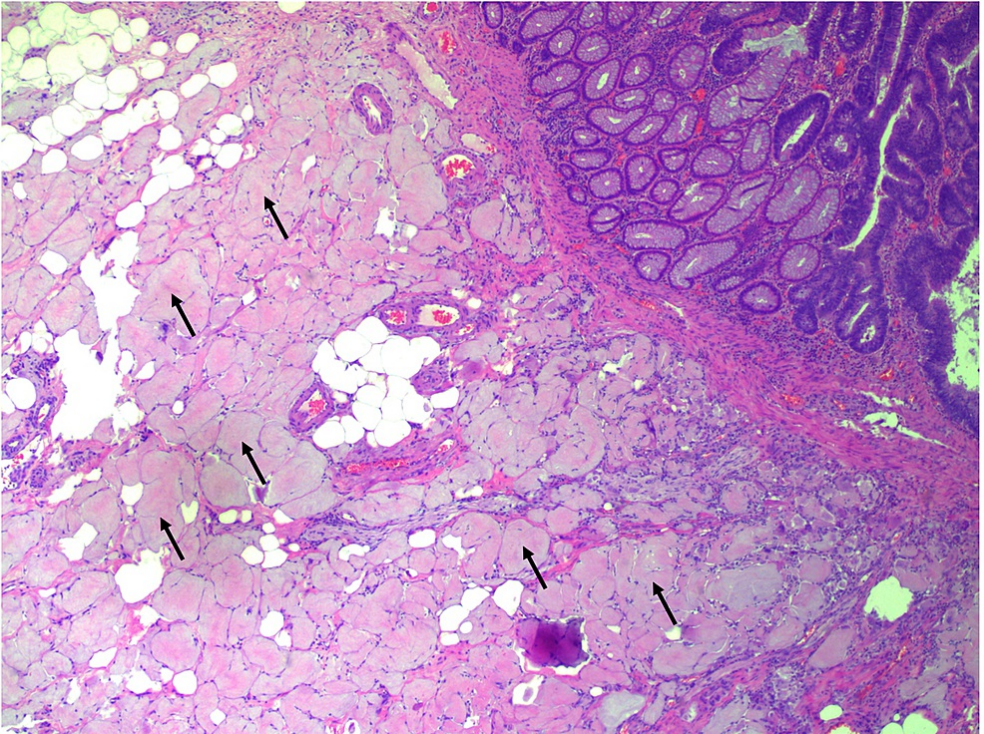
Pathology findings from biopsies showing involvement of gastric and esophagus squamous mucosa by deposition of an eosinophilic amorphous material with accompanying chronic inflammation and reactive epithelial changes.

Biopsies of the proximal esophagus, distal esophagus, and stomach were taken during the EGD for further histopathological investigation and were all shown to have the presence of a dense eosinophilic amorphous material with chronic inflammation (Figure 3).

**Figure 4:**
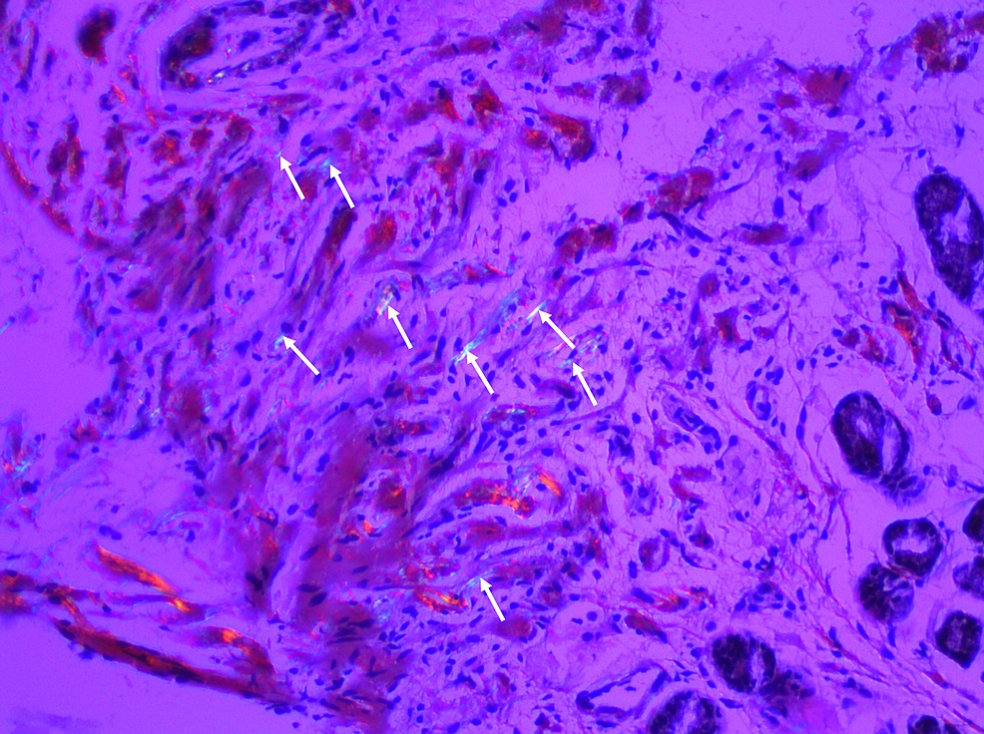
Congo red stain showing the amorphous material is congophilic and displays green birefringence under polarized light.

Further, histopathological studies showed that the amorphous material of the biopsy samples was congophilic and displayed a bright green birefringence under polarized light (Figure 4).

Therefore, these findings confirmed the presence of amyloidosis involving the proximal and distal esophagus as well as the gastric mucosa with chronic inflammation and reactive epithelial changes. Aside from revealing a potential cause of our patient’s dysphagia, the patient was referred to hematology/oncology specialists to investigate potential etiologies of our patient’s amyloidosis and to exclude a bone marrow disorder, such as multiple myeloma or plasma cell dyscrasias, in order to best determine the treatment course for the underlying disease [5]. 

## Discussion

The incidence of amyloidosis is difficult to characterize due to its variety of clinical presentations and the fact that only symptomatic patients are generally investigated. For example, previous studies have shown that gastric involvement occurs in 12% of cases by autopsy, with only 1% being symptomatic [4,5]. Further, the prevalence of amyloidosis with esophageal involvement has been reported to be within the ranges of 13% in a radiology study to 22% from an autopsy series [7]. However, the symptomatic presentation of these cases is most often limited to symptoms related to gastric reflux, making our patient’s presentation of dysphagia a unique case. It is important to note that the endoscopic findings in our case do not directly reveal a specific pathological presentation of amyloidosis; rather, they support the sentiment that GI amyloidosis consists of largely nonspecific endoscopic manifestations. For example, previous studies have found that the presentation of GI amyloidosis is highly variable, consisting of friable mucosa with erosions, ulcers, submucosal hematomas, or mucosal thickening [8]. Due to the variability of endoscopic findings for amyloidosis in the GI tract coupled with the presentation of nonspecific symptoms, clinicians should have a high degree of clinical suspicion when performing endoscopy as the diagnosis can be confirmed by histopathological studies. Furthermore, there are several underlying pathophysiological mechanisms of this patient’s unique clinical presentation of dysphagia. First, since amyloidosis is predominantly located in the muscularis mucosa, submucosa, and muscularis propria, this deposition of amyloid between muscle fibers in the esophagus could cause pressure atrophy, leading to a significant cause of dysmotility. Second, the deposition of amyloid in the submucosal vessels could hinder blood flow, thus leading to deterioration of the myenteric plexus function [9]. Third, amyloid deposition within the muscularis propria and submucosa could directly impact the Auerbach’s and Meissner’s plexuses throughout the GI tract, leading to various neurological presentations such as a dilated and atonic esophagus with diminished peristalsis and narrowing, presenting as a clinical picture similar to achalasia [9,10]. 

## Conclusions

As most cases of GI amyloidosis occur in the small bowel and liver presenting with cirrhotic sequelae or GI bleeding, our case report contributes a rare manifestation of dysphagia secondary to GI amyloidosis in the proximal and distal esophagus as well as in the gastric mucosa. Given the abnormal laboratory findings coupled with incidental findings of amyloidosis in several locations in this case, early detection and further characterization of the etiology is imperative in determining the treatment course and improving outcomes for these rare patients. By virtue of this case report, we hope to encourage clinicians to consider and report rare presentations of amyloidosis involvement in the GI tract. 
